# Cholesterol-modified prognostic nutritional index: a novel nutritional biomarker for prevalent diabetic retinopathy in patients with type 2 diabetes mellitus

**DOI:** 10.3389/fnut.2026.1942512

**Published:** 2026-09-24

**Authors:** Shimeng Huang, Li Wang, Cong Zhang, Ruoxuan Zheng, Xiaotong Sun, Jingjing Li, Kexin Cui, Yihan Wang, Rui Hou, Siyu Dong, Shuang Wu, Tianpeng Zhang, Xinyu Zhang, Chengye Xu, Hongyu Kuang

**Affiliations:** Department of Endocrinology, The First Affiliated Hospital of Harbin Medical University, Harbin, China

**Keywords:** cholesterol-modified prognostic nutritional index, diabetic retinopathy, LASSO regression, nomogram model, type 2 diabetes mellitus

## Abstract

**Purpose:**

To investigate the association and dose–response relationship between the cholesterol-modified prognostic nutritional index (CPNI) and diabetic retinopathy (DR) in patients with type 2 diabetes mellitus (T2DM), and to evaluate its utility in identifying prevalent DR.

**Methods:**

In this retrospective cross-sectional study, 1,750 patients with T2DM (654 with DR and 1,096 without DR) were enrolled. Multivariable logistic regression, subgroup, trend, and restricted cubic spline analyses were performed to evaluate the association between CPNI and prevalent DR. A nomogram for identifying prevalent DR was developed using variables selected by least absolute shrinkage and selection operator regression and multivariable logistic regression. Model performance was evaluated using discrimination, calibration, bootstrap internal validation, incremental predictive performance, and decision curve analysis.

**Results:**

Higher CPNI levels were independently associated with prevalent DR (OR = 1.024, 95% CI: 1.012–1.037, *p* < 0.001). A significant positive linear dose–response relationship between CPNI and prevalent DR was observed, with no evidence of nonlinearity in the RCS analyses. Subgroup analyses demonstrated stronger associations among participants with BMI < 25 kg/m^2^ and those with a history of alcohol consumption. A nomogram incorporating disease duration, using insulin, neutrophil, ALT, HbA1c, and CPNI demonstrated acceptable discrimination (apparent AUC = 0.724; optimism-corrected AUC = 0.715), good calibration, and favorable clinical utility.

**Conclusion:**

CPNI was independently and positively associated with prevalent DR in patients with T2DM and exhibited a linear dose–response relationship. A nomogram incorporating CPNI demonstrated acceptable performance for identifying prevalent DR.

## 1 Introduction

Diabetes has become a major global public health challenge, with its prevalence continuing to rise. In 2024, the prevalence of diabetes among adults aged 20–79 years worldwide was 11.11%, and it is projected to increase to 12.96% by 2050 in this age group (1, 2). Type 2 diabetes mellitus (T2DM) is the most common form of diabetes, accounting for approximately 90–95% of all cases (3). In addition to its long-term detrimental effects on health, T2DM can lead to a range of chronic complications that substantially impair quality of life and impose considerable societal and healthcare burdens. Among these complications, diabetic retinopathy (DR) is one of the most prevalent microvascular disorders, affecting nearly one-third of individuals with diabetes and representing a leading cause of visual impairment and blindness among adults worldwide (4, 5). The pathological features of DR include retinal microvascular abnormalities, tissue ischemia, neovascularization, and disruption of the blood–retinal barrier, ultimately resulting in irreversible visual loss (6, 7). Therefore, identifying factors associated with DR may be clinically relevant for early recognition and management of affected individuals.

Accumulating evidence suggests that nutritional and inflammatory status play pivotal roles in the development and progression of diabetes and its complications. The prognostic nutritional index (PNI), for example, has been extensively investigated in various T2DM-related complications and is considered an important predictor of diabetic peripheral neuropathy, diabetic nephropathy, and DR (8–11). Based on the traditional PNI, the cholesterol-modified prognostic nutritional index (CPNI) was initially developed in breast cancer research (12). Comprising total cholesterol, serum albumin, and lymphocyte count, CPNI has demonstrated favorable predictive performance in risk assessment and prognostic evaluation for conditions such as cancer and primary biliary cholangitis (12–14). Nevertheless, the role of CPNI in T2DM and its complications—particularly its clinical relevance in DR—has not yet been adequately investigated.

This study aimed to investigate the association between the CPNI and DR in patients with T2DM, and to develop a CPNI-based nomogram for identifying prevalent DR. The findings may provide evidence regarding the potential utility of nutritional-inflammatory status in DR screening and risk stratification.

## 2 Methods

### 2.1 Study design and participants

This study was designed as a retrospective cross-sectional observational study conducted at the First Affiliated Hospital of Harbin Medical University. In accordance with the Declaration of Helsinki, the study protocol was approved by the Ethics Committee of the First Affiliated Hospital of Harbin Medical University. Clinical data were collected from patients with T2DM who were hospitalized in the Department of Endocrinology between July 2024 and July 2025. The inclusion criteria were as follows: (1) adults aged ≥18 years who met the diagnostic criteria for T2DM; (2) availability of complete laboratory examination data; and (3) completion of optical coherence tomography angiography (OCTA) examination. The exclusion criteria included: (1) other types of diabetes; (2) active or chronic inflammatory conditions, active infections, autoimmune diseases, or acute/chronic renal or hepatic diseases; (3) a history of malignant tumors; (4) the presence of other severe ophthalmic diseases; and (5) pregnancy, incomplete clinical data, or inability to cooperate with examinations.

### 2.2 Definition of DR

DR assessment was performed during hospitalization using a standardized ophthalmologic protocol that included fundus photography and optical coherence tomography angiography (OCTA) for all participants. DR was defined by the presence of characteristic retinal lesions in either eye according to the Early Treatment Diabetic Retinopathy Study (ETDRS) criteria. The retinal images were independently evaluated by two experienced ophthalmologists from the First Affiliated Hospital of Harbin Medical University. When discrepancies arose between the two assessments, a third senior ophthalmologist independently reviewed the images, and the final diagnosis was determined by consensus (15). Importantly, the classification of DR was based on ophthalmologic findings and was not determined according to the laboratory measurements analyzed in this study.

### 2.3 Clinical and laboratory data collection

All patients were admitted between July 2024 and July 2025. Demographic characteristics and laboratory data were obtained from electronic medical records and outpatient documentation. Collected demographic and clinical variables included gender, age, disease duration, smoking history, alcohol consumption history, body mass index (BMI), history of using insulin, taking hypoglycemic drugs and history of cardiovascular disease. For laboratory assessments, fasting venous blood samples were collected before 8:00 a.m. and analyzed using standardized methods. Glycated hemoglobin (HbA1c) was measured by high-performance liquid chromatography. Fasting insulin levels were determined using radioimmunoassay. Enzymatic methods were used to assess alanine aminotransferase (ALT), aspartate aminotransferase (AST), albumin, fasting plasma glucose, total cholesterol, triglycerides, low-density lipoprotein cholesterol (LDL-C), high-density lipoprotein cholesterol (HDL-C), urea, uric acid (UA), and creatinine (Cr). Complete blood counts, including neutrophil, lymphocyte, and monocyte counts, were measured using an automated hematology analyzer (Sysmex XT-2000i, SYSMEX, Japan). CPNI was calculated using the following formula: CPNI = 4.8 × total cholesterol (mmol/L) − 1.5 × albumin (g/L) − 7.7 × lymphocyte count (×10^9^/L) + 126 (12–14).

### 2.4 Statistical analysis

All statistical analyses were performed using SPSS version 27.0, EmpowerStats version 2.0, Python version 3.8.13, and R version 4.3.3. Descriptive statistics were first used to summarize the study population. Continuous variables with a normal distribution are presented as mean ± standard deviation (SD) and were compared using the independent-samples *t* test. Continuous variables with a non-normal distribution are expressed as median (interquartile range, IQR) and were compared using the Mann–Whitney U test. Categorical variables are presented as frequencies and percentages and were compared using the chi-square test.

To develop a parsimonious model for identifying prevalent DR in patients with T2DM, all candidate variables were entered simultaneously into a least absolute shrinkage and selection operator (LASSO) regression model, including 27 demographic characteristics, clinical variables, and laboratory parameters, such as CPNI, total cholesterol, lymphocyte count, serum albumin, and other relevant variables. Variable selection was performed using 10-fold cross-validation, and the λ1se criterion was adopted to obtain a more parsimonious and stable set of selected variables. LASSO regression was used for variable selection in the subsequent development of the nomogram for identifying prevalent DR.

Univariate and multivariable logistic regression analyses were conducted to evaluate the association between CPNI and DR. The multivariable model was adjusted for gender, age, BMI, and the six non-zero variables selected by LASSO regression. The same covariate adjustment strategy was applied in the multivariable analyses, subgroup analyses, and restricted cubic spline (RCS) analyses. Participants were further categorized into quartiles according to CPNI levels, and trend tests were performed to assess the dose–response relationship between CPNI and the likelihood of prevalent DR. In addition, RCS analysis was conducted to explore potential nonlinear associations between CPNI and DR. Subgroup analyses and interaction tests were performed to evaluate potential effect modification. For subgroup analyses stratified by age, BMI, disease duration, using insulin, and HbA1c level, the corresponding stratification variable was excluded from the adjustment model, whereas all remaining prespecified covariates were retained. For all other subgroup analyses, the full set of prespecified covariates was included for adjustment. A nomogram for identifying DR was subsequently developed based on variables that remained statistically significant in the multivariable logistic regression analysis. Overall model fit was evaluated using the −2 log-likelihood, Akaike information criterion (AIC), and Bayesian information criterion (BIC). Model discrimination was assessed using receiver operating characteristic (ROC) curves and the area under the curve (AUC). Bootstrap internal validation with 1,000 resamples was performed to obtain optimism-corrected AUC estimates. The incremental discrimination and reclassification performance after incorporation of CPNI was evaluated using the integrated discrimination improvement (IDI) and net reclassification improvement (NRI). Model calibration was assessed using calibration curves, the Brier score, and the Hosmer–Lemeshow goodness-of-fit test. Clinical utility was evaluated through decision curve analysis (DCA). Spearman correlation analysis was additionally performed to examine the relationships between CPNI and other clinical indicators. All statistical tests were two-sided, and a *p* value <0.05 was considered statistically significant.

## 3 Results

### 3.1 Patient characteristics

A total of 1,750 patients with T2DM were included in this study, and their baseline clinical and laboratory characteristics are summarized in Table 1. Significant differences were observed between the DR and NDR groups in age, disease duration, BMI, history of hypertension, using insulin, neutrophil, fasting blood glucose, ALT, AST, LDL-C, albumin, urea, Cr, HbA1c, fasting insulin, and CPNI (*p* < 0.05).

**Table 1:** The baseline characteristics of the study population.

Variables	Non-diabetic retinopathy (*N* = 1,096)	Diabetic retinopathy (*N* = 654)	*p* value
Age (years)	55.000 (45.000–63.000)	56.500 (50.000–64.000)	<0.001***
Disease duration (years)	5.000 (1.000–10.000)	10.000 (5.000–18.000)	<0.001***
BMI (kg/m^2^)	25.551 (23.434–28.040)	25.279 (23.323–27.242)	0.039*
Gender, *n* (%)			0.447
Female	434 (39.599%)	247 (37.768%)	
Male	662 (60.401%)	407 (62.232%)	
Hypertension, *n* (%)			0.001***
No	644 (58.759%)	333 (50.917%)	
Yes	452 (41.241%)	321 (49.083%)	
Cardiovascular disease, *n* (%)			0.208
No	895 (81.661%)	518 (79.205%)	
Yes	201 (18.339%)	136 (20.795%)	
Using insulin, *n* (%)			<0.001***
No	643 (58.668%)	199 (30.428%)	
Yes	453 (41.332%)	455 (69.572%)	
Taking hypoglycemic drugs, *n* (%)			0.882
No	280 (25.547%)	165 (25.229%)	
Yes	816 (74.453%)	489 (74.771%)	
Smoking, *n* (%)			0.447
No	801 (73.084%)	467 (71.407%)	
Yes	295 (26.916%)	187 (28.593%)	
Alcohol, *n* (%)			0.234
No	863 (78.741%)	499 (76.300%)	
Yes	233 (21.259%)	155 (23.700%)	
Neutrophil (10^9^/L)	3.480 (2.830–4.322)	3.650 (2.960–4.560)	0.002**
Lymphocyte (10^9^/L)	1.935 (1.578–2.370)	1.890 (1.530–2.317)	0.128
Monocyte (10^9^/L)	0.430 (0.350–0.520)	0.440 (0.360–0.530)	0.190
Fasting blood glucose (mmol/L)	8.125 (6.800–10.220)	8.700 (6.703–11.138)	0.016*
Total cholesterol (mmol/L)	4.800 (3.970–5.600)	4.855 (4.060–5.750)	0.073
Triglyceride (mmol/L)	1.760 (1.200–2.802)	1.795 (1.200–2.897)	0.635
HDL-C (mmol/L)	1.100 (0.960–1.300)	1.100 (0.960–1.300)	0.594
LDL-C (mmol/L)	2.990 (2.270–3.680)	3.090 (2.430–3.770)	0.012*
ALT (U/L)	23.000 (16.595–36.800)	19.200 (14.200–27.500)	<0.001***
AST (U/L)	20.700 (16.400–28.425)	18.800 (15.400–23.800)	<0.001***
Albumin (g/L)	42.850 (40.600–45.500)	42.100 (39.615–44.400)	<0.001***
Urea (mmol/L)	5.470 (4.615–6.560)	5.870 (4.950–7.123)	<0.001***
Cr (μmol/L)	63.000 (53.000–74.225)	65.000 (53.225–78.000)	0.021*
UA (μmol/L)	328.050 (272.875–396.975)	335.200 (275.400–409.775)	0.340
HbA1c (%)	8.300 (7.175–9.725)	8.800 (7.600–10.300)	<0.001***
HbA1c (mmol/mol)	67.213 (54.918–82.787)	72.678 (59.563–89.071)	<0.001***
Fasting Insulin (μU/ml)	11.095 (7.345–15.908)	10.145 (6.510–15.660)	0.015*
CPNI	69.637 (63.677–75.180)	71.668 (65.673–77.209)	<0.001***

Data are expressed as mean ± standard deviation, percentage, or median (IQR); *p*-values were compared using independent *t*-tests, Mann–Whitney U tests, or chi-square tests. Significance was set at * *p* < 0.05, ** *p* < 0.01, *** *p* < 0.001. DR, diabetic retinopathy, BMI, body mass index, HDL-C, high-density lipoprotein cholesterol, LDL-C, low-density lipoprotein cholesterol, ALT, Alanine Aminotransferase, AST, Aspartate Aminotransferase; Cr, Creatinine, UA, uric acid, HbA1c, glycated hemoglobin, CPNI, Cholesterol-modified prognostic nutritional index.

### 3.2 Key variable selection using LASSO regression

LASSO regression is a linear regression technique that performs feature selection by introducing an L1 regularization penalty, enabling automatic identification of the most predictive variables through coefficient shrinkage while effectively preventing model overfitting (16). Six variables with non-zero coefficients were selected by LASSO regression, including disease duration, using insulin, neutrophil, ALT, HbA1c, and CPNI (Figure 1).

**Figure 1:**
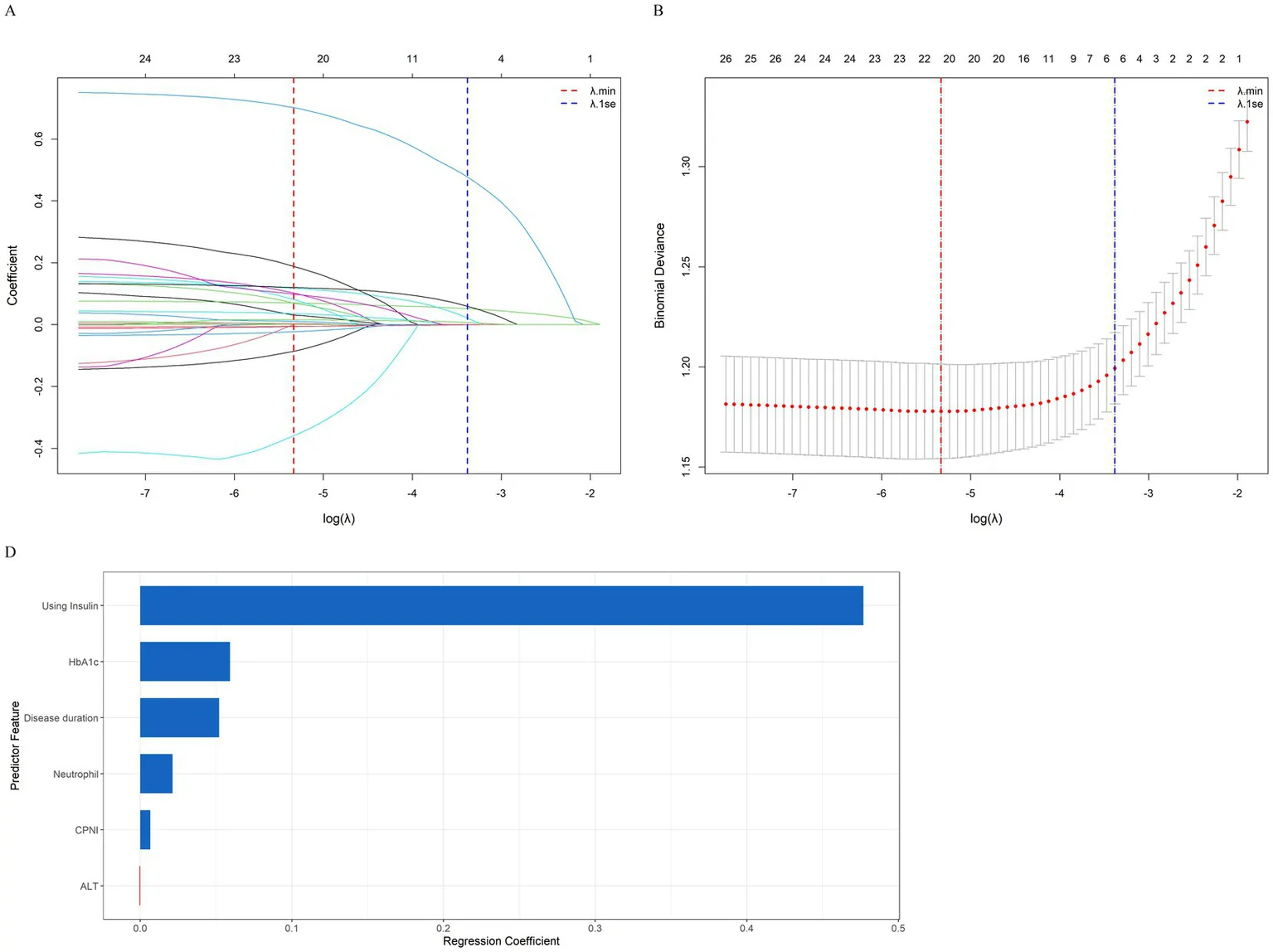
Variable selection using LASSO regression. **(A)** Coefficient profiles of candidate variables; **(B)** Selection of the optimal λ value using 10-fold cross-validation; **(C)** Coefficients of the variables selected by the Lasso regression model. Abbreviations are listed in Table 1.

### 3.3 Factors associated with DR and the dose–response relationship between CPNI and DR

To investigate factors associated with DR and the relationship between CPNI and DR, variables selected by LASSO regression were included in subsequent analyses, with age, gender, and BMI additionally incorporated based on their clinical relevance. Univariate logistic regression analyses were first performed for all included variables (Figure 2A). Age, BMI, disease duration, using insulin, neutrophil, ALT, HbA1c, and CPNI were significantly associated with DR (all *p* < 0.05), whereas gender was not (*p* = 0.447). As a continuous variable, CPNI was positively associated with DR (OR = 1.031, 95% CI: 1.020–1.042, *p* < 0.001). All included variables were subsequently entered into the multivariable logistic regression model (Figure 2B). After adjustment for potential confounders, CPNI remained independently associated with DR (OR = 1.024, 95% CI: 1.012–1.037, *p* < 0.001), indicating that higher CPNI levels were associated with greater odds of prevalent DR.

**Figure 2:**
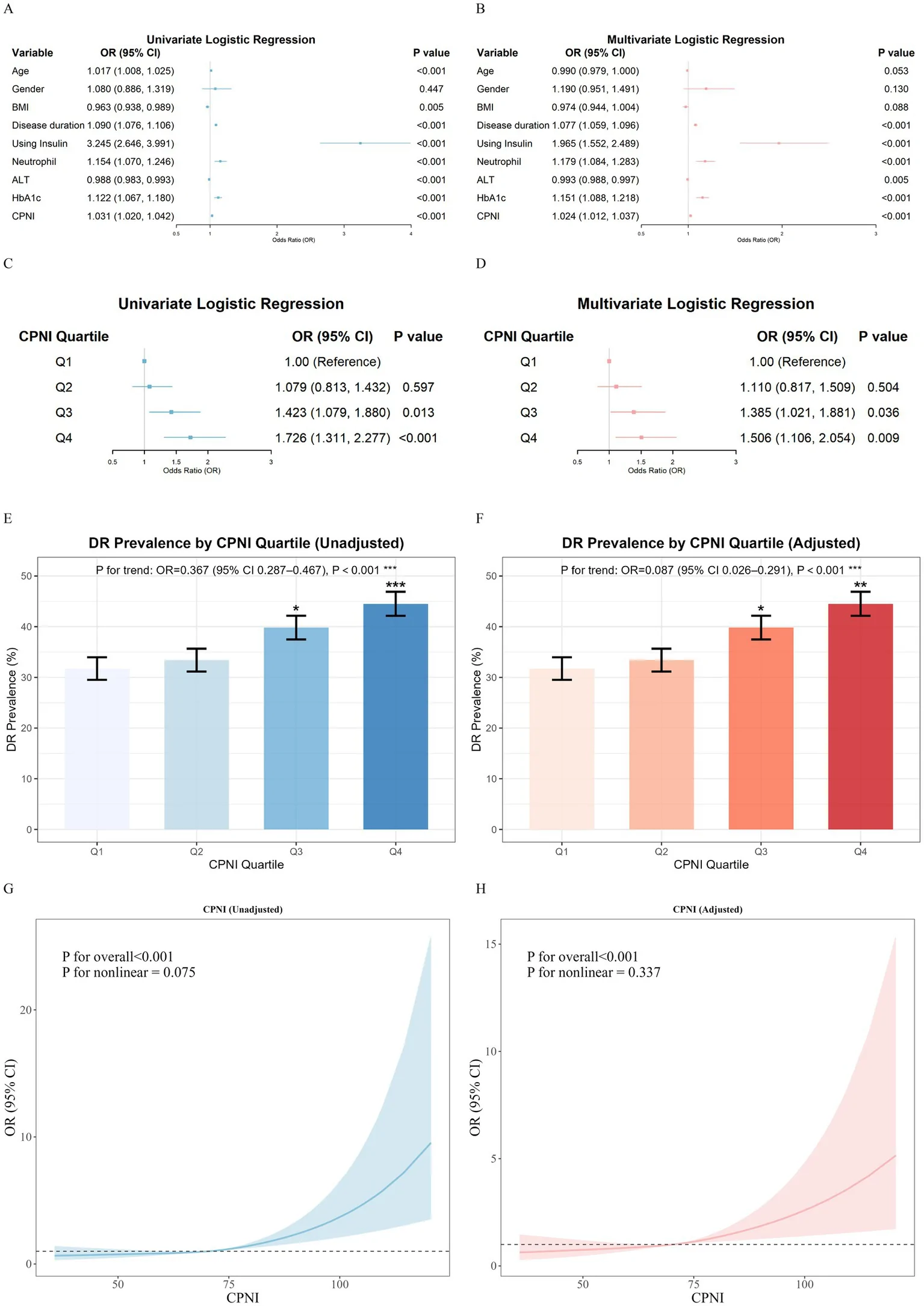
Association and dose–response relationship between CPNI and prevalent DR. **(A)** Univariate logistic regression analysis with CPNI as a continuous variable; **(B)** Multivariable logistic regression analysis with CPNI as a continuous variable; **(C)** Univariate logistic regression analysis with CPNI categorized into quartiles; **(D)** Multivariable logistic regression analysis with CPNI categorized into quartiles; **(E)** Test for linear trend in the crude model; **(F)** Test for linear trend in the adjusted model; **(G)** Restricted cubic spline (RCS) analysis in the crude model; **(H)** Restricted cubic spline (RCS) analysis adjusted for age, gender, BMI, disease duration, using insulin, neutrophil, ALT, and HbA1c. * *p* < 0.05, ** *p* < 0.01, *** *p* < 0.001. OR, odds ratio; CI, confidence interval. Abbreviations are listed in Table 1.

To further evaluate the dose–response relationship between CPNI and DR, participants were categorized into quartiles according to CPNI, with the lowest quartile (Q1) serving as the reference group. Univariate and multivariable logistic regression analyses were then performed. In the univariate analysis (Figure 2C), compared with participants in Q1, those in Q3 (OR = 1.423, 95% CI: 1.079–1.880, *p* = 0.013) and Q4 (OR = 1.726, 95% CI: 1.311–2.277, *p* < 0.001) had significantly higher odds of DR, whereas no significant difference was observed between Q2 and Q1 (OR = 1.079, 95% CI: 0.813–1.432, *p* = 0.597). Trend analysis demonstrated that the odds of DR increased progressively across increasing CPNI quartiles (P for trend <0.001) (Figure 2E). After adjustment for age, gender, BMI, disease duration, using insulin, neutrophil, ALT, and HbA1c, this association persisted (Figure 2D). Compared with Q1, participants in Q3 (OR = 1.385, 95% CI: 1.021–1.881, *p* = 0.036) and Q4 (OR = 1.506, 95% CI: 1.106–2.054, *p* = 0.009) had higher odds of prevalent DR, whereas no significant difference was observed between Q2 and Q1 (OR = 1.110, 95% CI: 0.817–1.509, *p* = 0.504) (Figure 2D). The positive trend across increasing CPNI quartiles remained statistically significant after multivariable adjustment (P for trend <0.001), further supporting a dose–response relationship between CPNI and DR (Figure 2F).

Restricted cubic spline (RCS) analysis was further performed to assess the overall and potential nonlinear associations between CPNI and DR. In the unadjusted model (Figure 2G), the overall association between CPNI and DR was statistically significant (P for overall < 0.001), whereas no significant nonlinear association was observed (P for nonlinear = 0.075). After adjustment for age, gender, BMI, disease duration, using insulin, neutrophil, ALT, and HbA1c (Figure 2H), the overall association remained significant (P for overall < 0.001), while the test for nonlinearity remained non-significant (P for nonlinear = 0.337). Collectively, these findings indicate that CPNI was positively and linearly associated with DR, with no evidence of a significant nonlinear relationship.

### 3.4 Subgroup analysis and interaction effects

To further evaluate the consistency of the association between CPNI and DR across different populations, subgroup analyses and interaction tests were performed according to gender, disease duration, age, BMI, hypertension, using insulin, HbA1c, fasting blood glucose, smoking, and alcohol (Figure 3). Significant interactions were observed only for BMI (P for interaction = 0.028) and alcohol consumption status (P for interaction = 0.047). The association between CPNI and DR was more pronounced with BMI < 25 kg/m^2^ (OR = 1.040, 95% CI: 1.022–1.059) and among those with a history of alcohol consumption (OR = 1.048, 95% CI: 1.018–1.080). No statistically significant interactions were detected in the remaining subgroups (all P for interaction > 0.05). Overall, the direction of the association between CPNI and DR remained consistent across subgroups, with no substantial heterogeneity observed.

**Figure 3:**
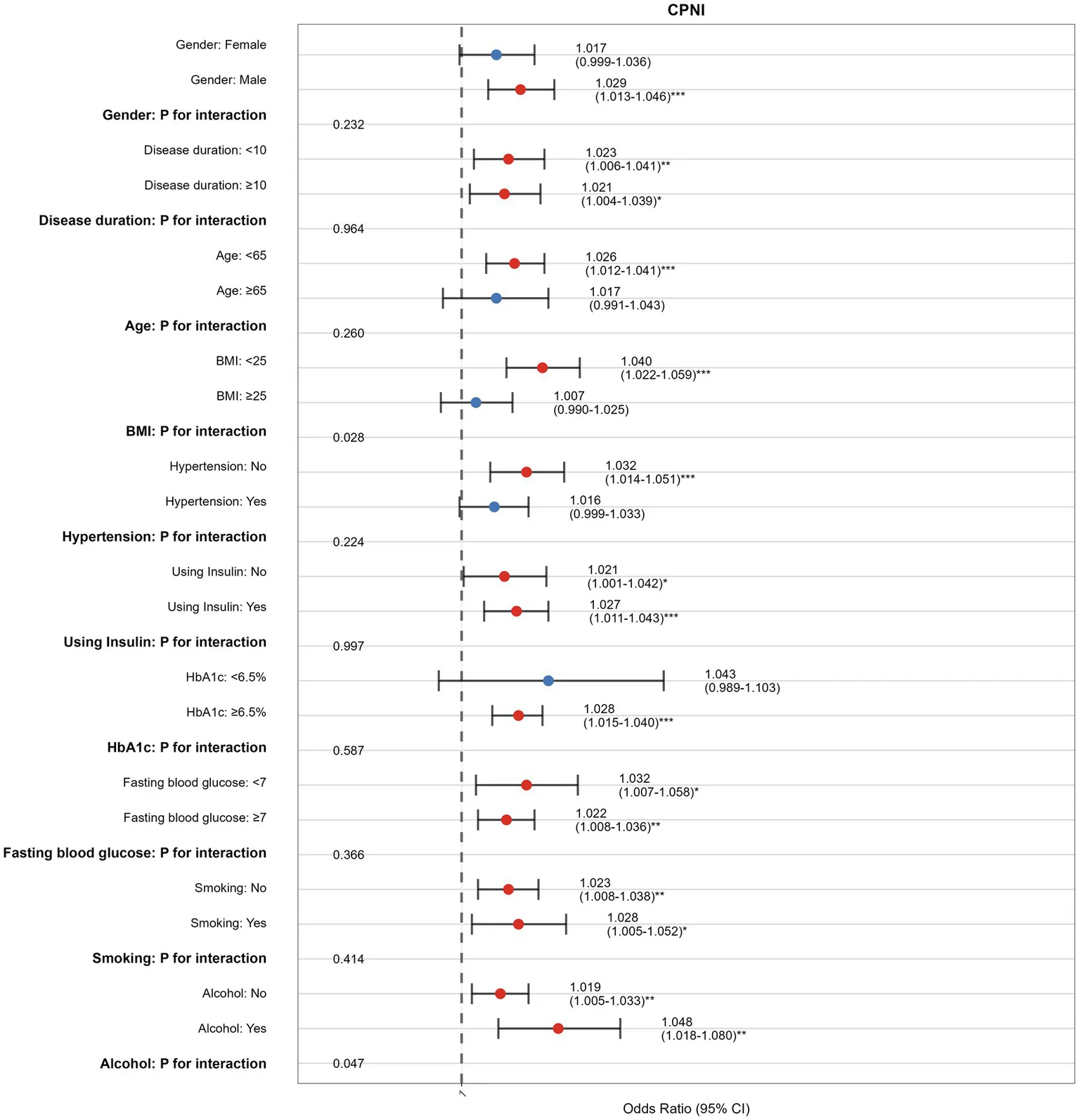
Subgroup and interaction analyses of the association between CPNI and prevalent DR. All subgroup and interaction analyses were adjusted for age, gender, BMI, disease duration, using insulin, neutrophil, ALT, and HbA1c. In analyses stratified by age, BMI, disease duration, using insulin, and HbA1c, the corresponding stratification variable was not included in the adjustment model. * *p* < 0.05, ** *p* < 0.01, *** *p* < 0.001. OR, odds ratio; CI, confidence interval. Abbreviations are listed in Table 1.

### 3.5 Construction and internal validation of a nomogram for identifying prevalent DR

Based on the variables independently associated with prevalent DR in the multivariable logistic regression analysis, including disease duration, using insulin, neutrophil, ALT, HbA1c, and CPNI, a nomogram for identifying prevalent DR was constructed (Figure 4A). Supplementary Table 1 and Figure 4 summarize the model fitting, discrimination, and calibration performance of the basic clinical model and the CPNI-incorporated nomogram.

**Figure 4:**
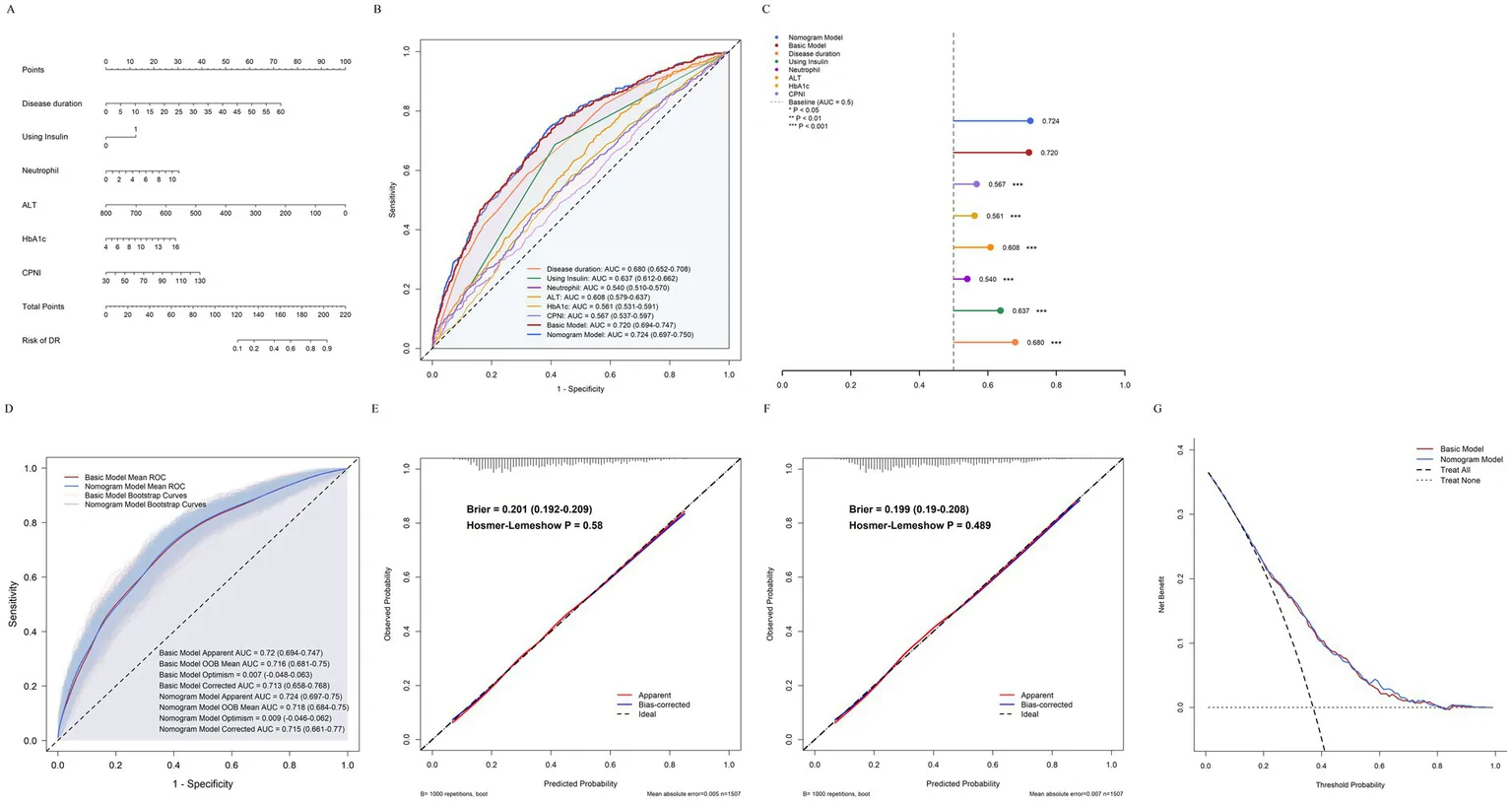
Development and internal validation of the nomogram for identifying prevalent DR. **(A)** Nomogram for identifying prevalent DR; **(B)** Receiver operating characteristic (ROC) curves for the individual variables, the basic model, and the nomogram; **(C)** DeLong test comparing the AUCs of the individual variables and the basic model with that of the nomogram. The gray dashed line indicates an AUC of 0.5; **(D)** Bootstrap-corrected ROC curves of the basic model and the nomogram following internal validation; **(E)** Calibration curve of the basic model; **(F)** Calibration curve of the nomogram model; **(G)** Decision curve analysis comparing the net clinical benefit of the basic model and the nomogram. ROC, receiver operating characteristic; AUC, area under the receiver operating characteristic curve. Abbreviations are listed in Table 1.

Compared with the basic model, the nomogram showed lower values for the −2 log-likelihood (1764.40 vs. 1775.05), AIC (1776.40 vs. 1785.05), and BIC (1813.63 vs. 1816.96), indicating improved overall model fit following the incorporation of CPNI. The apparent AUC of the nomogram was 0.724 (95% CI: 0.697–0.750), which was slightly higher than that of the basic model (0.720, 95% CI: 0.694–0.747) (Figure 4B). After 1,000-bootstrap internal validation, the optimism-corrected AUC was 0.715 (95% CI: 0.661–0.770) for the nomogram and 0.713 (95% CI: 0.658–0.768) for the basic model (Figure 4D). However, the difference in AUC between the two models was not statistically significant according to the DeLong test (Figure 4C).

Incremental discrimination analysis showed an IDI of 0.006 (95% CI: 0.002–0.010), with the confidence interval entirely above zero, indicating a small but statistically significant improvement in continuous risk discrimination after incorporation of CPNI. In contrast, the NRI was 0.071 (95% CI: −0.035–0.183), with the confidence interval crossing zero, suggesting that the addition of CPNI did not significantly improve risk reclassification.

Regarding calibration, the nomogram achieved a slightly lower Brier score than the basic model (0.199 [95% CI: 0.190–0.208] vs. 0.201 [95% CI: 0.192–0.209]) (Figures 4E,F). The Hosmer–Lemeshow goodness-of-fit test was non-significant for both models (nomogram: *p* = 0.489; basic model: *p* = 0.580), indicating good agreement between the predicted probabilities and the observed outcomes (Figures 4E,F). Decision curve analysis further showed that the nomogram provided a positive net clinical benefit across a wide range of threshold probabilities (Figure 4G).

### 3.6 Correlations between CPNI and other clinical indicators

Spearman correlation analysis was performed to explore the relationships between CPNI and other clinical indicators (Supplementary Figure 1). CPNI was positively correlated with age, disease duration, fasting blood glucose, HbA1c, triglycerides HDL-C, LDL-C, total cholesterol, using insulin, and urea (all *p* < 0.05). In contrast, CPNI was negatively correlated with ALT, AST, albumin, BMI, cardiovascular disease, fasting insulin, gender, lymphocyte, monocyte, neutrophil, smoking, taking hypoglycemic drugs, and UA (all *P* < 0.05). No significant correlations were observed between CPNI and alcohol, Cr, or hypertension (all *p* > 0.05).

## 4 Discussion

To our knowledge, this is the first study to systematically evaluate the association between the CPNI and DR in patients with T2DM and to establish a CPNI-based nomogram for identifying prevalent DR. The principal findings were as follows: (1) CPNI was independently associated with DR after adjustment for multiple confounding factors; (2) a significant positive linear dose–response association was observed between CPNI and DR; and (3) a nomogram incorporating disease duration, using insulin, neutrophil, ALT, HbA1c, and CPNI showed acceptable discrimination, calibration, and internal validity for identifying prevalent DR.

Malnutrition and diabetes, along with its complications, are linked through complex bidirectional relationships. In individuals with diabetes, malnutrition may impair pancreatic β-cell health by limiting the availability of nutrients essential for their function, thereby weakening insulin secretory responses to glycemic stimuli, exacerbating dysregulation of glucose homeostasis, and promoting the onset and progression of diabetes (17). In populations with T2DM, patients with concomitant sarcopenia and malnutrition experience longer hospital stays and a higher one-year mortality risk compared with those who are well nourished and free of sarcopenia (18). Moreover, malnutrition may further accelerate the progression of diabetes-related microvascular and macrovascular complications. Epidemiological evidence suggests that malnutrition affects approximately 30% of the global population and is particularly prevalent among older adults with multiple comorbidities. In patients with diabetes, malnutrition and related nutritional risk remain frequently underestimated (19). Previous studies have reported that the prevalence of malnutrition among individuals with T2DM is approximately 33%, while the prevalence of nutritional risk is as high as 44%; malnutrition has been recognized as a critical determinant of adverse outcomes in this population (19, 20).

CPNI integrates three key indicators: total cholesterol, serum albumin, and lymphocyte count. It reflects the body’s nutritional–inflammatory–immune status from multiple dimensions, including nutritional reserves, inflammatory regulation, and immune function, and has potential clinical utility for disease assessment and stratification. Previous studies have shown that elevated total cholesterol is closely associated with retinal microvascular damage in DR, with significant positive correlations between total cholesterol levels and retinal ECE-1 (Pearson r = 0.888, 95% CI: 0.2735–0.9877, *p* = 0.018) and ET-1 expression (Pearson r = 0.815, 95% CI: 0.0088–0.9790, *p* = 0.048). This may be mechanistically linked to high cholesterol–induced systemic lipid metabolism disorder, which activates the ECE-1/ET-1 signaling pathway via oxidative stress and inflammatory responses, thereby exacerbating retinal endothelial dysfunction (21). Additionally, cholesterol-derived indicators, such as remnant cholesterol and the remnant cholesterol/high-density lipoprotein cholesterol ratio, have been identified as independent risk factors for DR (22, 23). Serum albumin, an important marker of nutritional reserves, plays a critical role in immune regulation, fluid homeostasis, and antioxidant processes. Albumin deficiency can exacerbate oxidative stress and inflammatory responses, and hypoalbuminemia has been associated with increased risks of cardiovascular disease, hyperlipidemia, and mortality, potentially aggravating oxidative damage indirectly through effects on cholesterol metabolism and liver function (24, 25). Studies have also confirmed that serum albumin and related ratios, such as the red blood cell distribution width/albumin ratio, are closely linked to DR risk (26, 27). Lymphocytes, as essential components of immune regulation, may contribute to local retinal inflammatory imbalance when reduced in number or function. Elevated monocyte-to-lymphocyte ratio (MLR) and neutrophil-to-lymphocyte ratio (NLR) can exacerbate retinal endothelial injury and promote DR progression, and have been significantly associated with DR risk (28, 29). Furthermore, systemic composite inflammatory indices differed across different stages of DR. Several systemic inflammatory markers, including NLR, MLR, and pan-immune-inflammation value, were significantly elevated in patients with proliferative diabetic retinopathy, whereas platelet-to-lymphocyte ratio and systemic immune-inflammation index levels were higher in patients with non-proliferative diabetic retinopathy. These findings suggest that systemic immune-inflammatory dysregulation may be involved in the development and progression of DR and that composite inflammatory indices derived from routinely available laboratory tests may serve as complementary tools for the assessment and monitoring of DR (30). Specific lymphocyte subsets, such as activated CD4^+^CD25^+^ T cells, can support retinal ganglion cell survival by regulating microglial and macrophage infiltration, partially delaying neurodegenerative changes in DR (31). Our study found that higher CPNI levels were significantly associated with greater odds of prevalent DR. This observation points to a link between impaired nutritional-inflammatory status and DR, although causal inference cannot be drawn given the cross-sectional study design.

Subgroup analyses indicated some heterogeneity in the association between CPNI and prevalent DR across different patient subgroups. Interaction analyses suggested that BMI and alcohol consumption may modulate this relationship. Among patients with BMI < 25 or those who consumed alcohol, higher CPNI levels were more strongly associated with prevalent DR, whereas this association was not statistically significant in individuals with BMI ≥ 25. These findings are biologically plausible. We hypothesize that, compared with individuals with higher BMI, the impact of nutritional imbalance on metabolic dysregulation may be more pronounced in those with lower BMI, thereby potentially strengthening the observed association between CPNI and prevalent DR. Additionally, alcohol intake may further impair nutrient absorption, metabolic homeostasis, and hepatic insulin sensitivity, exacerbating adverse metabolic consequences related to malnutrition. These results underscore the importance of considering individual metabolic characteristics and lifestyle factors when evaluating the relationship between nutrition–inflammation markers and prevalent DR, though the underlying mechanisms require further investigation.

In the present study, six variables, including using insulin, disease duration, HbA1c, neutrophil, CPNI, and ALT, were selected by LASSO regression and incorporated into a nomogram for the identification of prevalent DR. The original AUC was 0.724 and decreased to 0.715 after optimism correction using 1,000 bootstrap resamples, indicating acceptable discrimination. The model also showed good calibration, with a Brier score of 0.199 and no evidence of significant lack of fit according to the Hosmer–Lemeshow test (*p* = 0.489). Importantly, although CPNI remained independently associated with prevalent DR, its incremental value beyond the base clinical model was limited. The base model included using insulin, disease duration, HbA1c, neutrophil, and ALT. Adding CPNI increased the AUC by only 0.004, with no statistically significant difference by the DeLong test (*p* > 0.05). Although the IDI reached statistical significance, the magnitude of improvement was small (IDI = 0.006, 95% CI: 0.002–0.010), while the NRI was not statistically significant (NRI = 0.071, 95% CI: −0.035–0.183). Given that AUC, NRI, and IDI assess different aspects of model performance, these findings are not necessarily contradictory; however, the statistically significant IDI should not be interpreted as evidence of a substantial clinical benefit. DCA suggested a greater net benefit of the CPNI-based nomogram across part of the threshold probability range, but such findings are dependent on the clinical threshold and study population and do not directly demonstrate improved real-world screening efficiency or patient outcomes. Overall, these findings suggest that CPNI may provide some complementary information beyond traditional clinical variables, although its incremental contribution to model performance appears limited. Larger, prospective, multicenter studies with external validation are needed to determine whether CPNI can provide clinically meaningful value for DR identification and whether it has utility for predicting the future development of DR.

The main strengths of this study are as follows. First, to our knowledge, this is the first study to systematically demonstrate an independent association between CPNI and DR in patients with T2DM. Second, we developed a CPNI-based nomogram for identifying prevalent DR. Third, from the perspective of CPNI, we explored subgroup heterogeneity in the association with DR and identified BMI and alcohol consumption as potential effect modifiers, providing additional insights into the heterogeneity of the association between CPNI and prevalent DR. Fourth, multiple complementary statistical approaches were applied to validate the robustness of the findings, thereby enhancing the reliability of the results.

Several limitations should also be acknowledged. First, due to the cross-sectional design, causal relationships between CPNI and DR cannot be established, and the proposed nomogram should be interpreted as a tool for identifying prevalent DR rather than predicting future DR occurrence. Whether CPNI can predict incident DR remains unknown and warrants evaluation in prospective longitudinal studies. Second, the study population was recruited from a single center, which may have introduced selection bias and may limit the generalizability of the findings to broader and more diverse populations with T2DM. Third, information regarding lipid-lowering agents, antihypertensive medications, and other treatments that may influence cholesterol metabolism and inflammatory status was unavailable. Therefore, residual confounding cannot be completely excluded. Fourth, although CPNI integrates cholesterol, albumin, and lymphocyte count into a composite index, the relative contribution of each component to DR risk was not separately evaluated. Fifth, although the nomogram demonstrated moderate discrimination, with an AUC of 0.724, its practical clinical utility should be interpreted cautiously, particularly given the limited incremental improvement over the clinical model. Bootstrap resampling provided internal validation and optimism correction within the current study population but cannot establish the model’s performance in other populations. Therefore, future large-scale, prospective, multicenter studies involving diverse populations are warranted to assess the ability of CPNI to predict incident DR and to externally validate the clinical utility and generalizability of the proposed nomogram.

## 5 Conclusion

In conclusion, CPNI was independently and positively associated with prevalent DR in patients with T2DM, with evidence of a linear dose–response relationship. A nomogram incorporating CPNI and other clinical variables demonstrated acceptable performance for identifying prevalent DR. Further prospective multicenter studies are warranted to determine whether CPNI is associated with subsequent DR development and whether it provides additional value for future risk prediction.

## Data Availability

The original contributions presented in the study are included in the article/Supplementary material, further inquiries can be directed to the corresponding author.
