# Supplementary material for: Cholesterol-modified prognostic nutritional index: a novel nutritional biomarker for prevalent diabetic retinopathy in patients with type 2 diabetes mellitus
Source: Front Nutr. 2026 Sep 24;13:1942512. doi: 10.3389/fnut.2026.1942512 (PMC13645571; doi:10.3389/fnut.2026.1942512)
Supplement: Supplementary file 1 [file Supplementary_file_1.DOCX]

**Supplementary Table 1. Comparison of predictive performance among base model and full nomogram model for DR**

| Metric | Basic Model | Nomogram Model |
| --- | --- | --- |
| minus2LL | 1775.05 | 1764.4 |
| AIC | 1785.05 | 1776.4 |
| BIC | 1816.96 | 1813.63 |
| Apparent_AUC (95% CI) | 0.720 (0.694—0.747) | 0.724 (0.697—0.750) |
| Corrected_AUC (95% CI) | 0.713 (0.658—0.768) | 0.715 (0.661—0.770) |
| Brier (95% CI) | 0.201 (0.192—0.209) | 0.199 (0.190—0.208) |
| Hosmer–Lemeshow P value | 0.580 | 0.489 |
| Total NRI (95% CI) | — | 0.071 (-0.035—0.183) |
| Total IDI (95% CI) | — | 0.006 (0.002—0.010) |

Note: minus2LL = minus 2 log-likelihood; AIC = Akaike information criterion; BIC = Bayesian information criterion; AUC = area under the receiver operating characteristic curve; NRI = net reclassification index; IDI = integrated discrimination improvement; vs = versus; CI = confidence interval; NRI and IDI were only calculated for nested models (basic model vs. nomogram model with additional CPNI). *P < 0.05, **P < 0.01, ***P < 0.001. Abbreviations as shown in Table 1.

Supplementary Figure 1. Spearman correlation analysis between CPNI and clinical variables. *P < 0.05, **P < 0.01, ***P < 0.001. Abbreviations are listed in Table 1.
